# Systematic heterogenisation to improve reproducibility in animal studies

**DOI:** 10.1371/journal.pbio.3001629

**Published:** 2022-05-06

**Authors:** Patrick Remus Suman, Cilene Lino de Oliveira

**Affiliations:** 1 Institute of Biophysics Carlos Chagas Filho, Federal University of Rio de Janeiro, RJ, Brazil; 2 Department of Physiological Sciences, Center of Biological Sciences, Federal University of Santa Catarina–UFSC, Florianópolis–SC, Brazil

## Abstract

A recent study published in PLoS Biology investigated whether the systematic use of multiple experimenters boosts the reproducibility of behavioural assays in mice. This Primer explores study designs to investigate systematic heterogenization in single-or multi-laboratory settings.

## Teaser

A recent study published in *PLOS Biology* investigated whether the systematic use of multiple experimenters boosts the reproducibility of behavioural assays in mice. These findings open up prospects for solutions to reproducibility issues in animal research.

**Fig 1:**
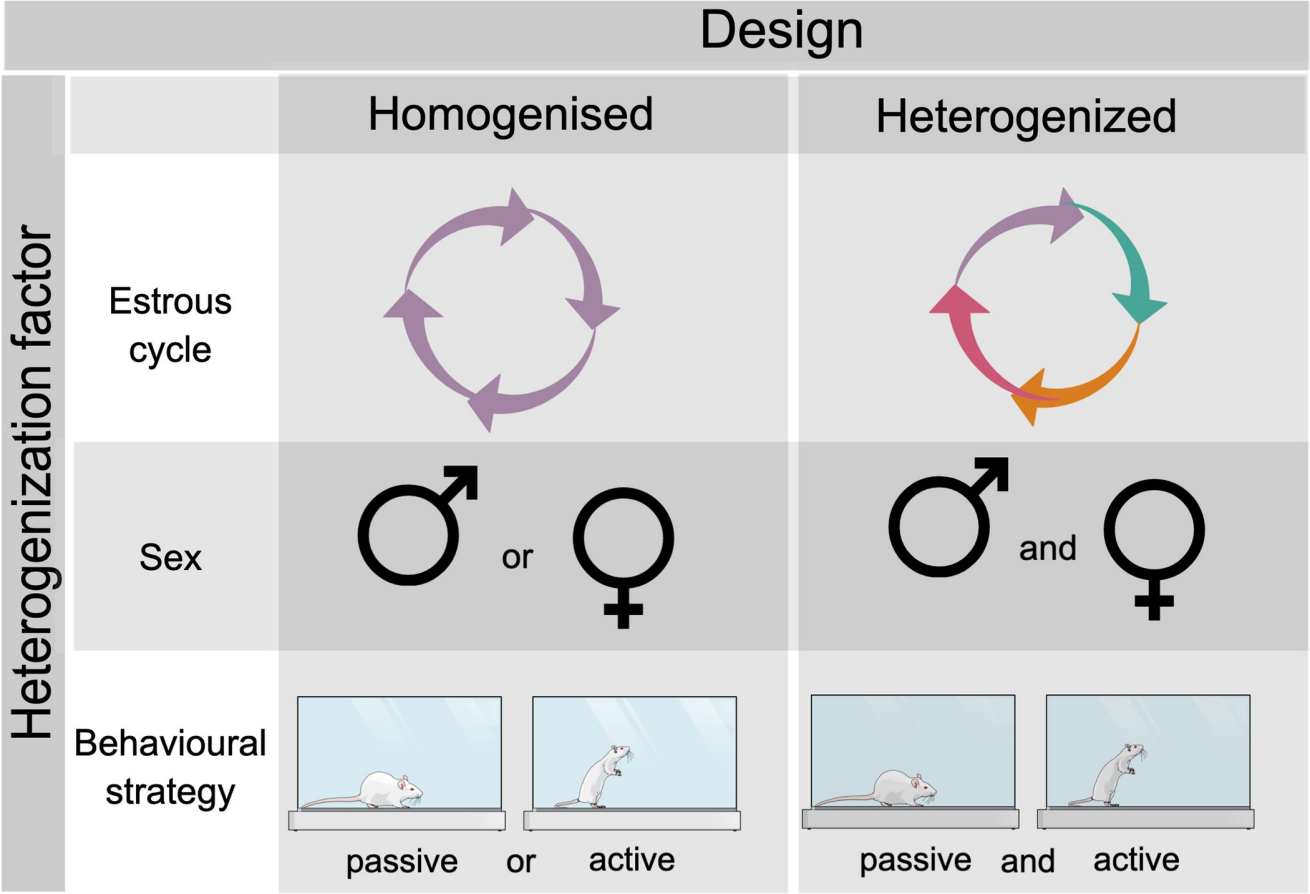
Design of hypothetical studies aiming to investigate systematic heterogenisation approaches to improve reproducibility in animal studies. Behavioural studies were replicated using single- or multilaboratory configurations, following homogenised and heterogenised designs. The estrous cycle, sex, or behavioural strategy can be used as a heterogenisation factor. Experimental groups in the homogenised design would be homogeneous (e.g., single estrous phase, single sex, and single behavioural strategy), while in the heterogenised design, groups would be heterogeneous (e.g., mixture of estrous phases, mixture of sexes, and mixture of behavioural strategies). The reproducibility of the studies was estimated by comparing the consistency of the outcomes within homogenised or heterogenised designs.

Reproducibility defies scientists in animal research. The cause of poor reproducibility ranges from technical issues to the unintended consequences of scientific practices (publication bias, perverse incentives, and so on) [1]. Additionally, the same species-specific cognitive and emotional systems that make laboratory animals useful for biological research bring variation to the studies [2]. Biology interacts with the environment adding variability layers to animal experiments [3]. The scientific community has pursued solutions to mitigate inconsistencies and avoid research waste in animal science [4–9]. A recent article published in *PLOS Biology* by von Kortzfleisch and colleagues [10] reported a strategy to minimise the incidence of contradictory results in mice behavioural assays, which was partially successful. Building upon this research [10], we conceived some hypothetical studies seeking to investigate systematic heterogenisation approaches to improve reproducibility in animal studies (Fig 1).

Scientists strive to keep their procedures standardised as much as possible, to eliminate variation, obtain accurate results in the long run, and extract the maximum information using a minimum number of animals. A homogenous population of animals (of the same sex, age, strain, etc.) kept under the same controlled conditions (food, water, temperature, humidity, etc.) tested simultaneously across experiments, preferentially by the same experimenters, are examples of standardisation. Nevertheless, biological variation precludes complete homogenisation of animal studies, making repeatability imperfect, even in the same laboratory over time [4]. Furthermore, homogenising laboratory conditions brings consistency in replica studies at the cost of generalisation, yielding results that are often idiosyncratic to a particular laboratory, which may damage reproducibility among laboratories [3,5,6]. In this context, systematic heterogenisation has emerged as a practical alternative to incomplete standardisation and a reasonable solution to the homogenisation–generalisation impasse.

Theoretically, adding a known source of variation to the experimental design boosts reproducibility once the portion of unknown variance in the study diminishes [5,6]. Proof-of-concept studies have found positive effects of systematic heterogenisation on reproducibility in some experimental settings [7,8], but not in others [9,10]. For example, Bodden and colleagues [7] observed in a simulation that the inclusion of 2 different testing times improved the reproducibility between replica experiments in the same laboratory. Likewise, von Kortzfleisch and colleagues [8] observed better reproducibility by splitting the experiment into several “mini-experiments” spread over different time points a few weeks apart in a single laboratory study. Contrastingly, heterogenised designs provide modest improvements in reproducibility across laboratories [9,10]. For example, systematically varying the age and cage enrichment of mice or the number of experimenters was insufficient to overcome the large variation between laboratories for most outcomes [9,10]. Experimenters, and other handlers of experimental animals, seem promising heterogenisation factors since they are distinctive elements in a study contributing to idiosyncratic results obtained in a laboratory. In this framework, results by Kortzfleisch and colleagues [10] showing that “experimenters” explained on average 5% of the experimental variation appear counterintuitive deserving a second look.

Homogeneous design used the same person as experimenter across the experiments in each laboratory (A, B, or C), whereas the heterogenised design included several experimenters within laboratories (A, B, and C) [10]. In the homogenous design, differences between the 2 strains of female mice (C57BL/6J-DBA/2N) varied in direction, magnitude, and statistical significance through laboratories for some outcomes. For example, rearing in a new cage varied from significantly higher in DBA/2N in “Lab A” to significantly higher in C57BL/6J in “Lab C,” while small difference was observed in “Lab B.” Time in the centre of the open field was significantly higher in C57BL/6J than in DBA/2N in laboratories A and C, while a small difference appears in “Lab B.” These discrepant results, leading to opposing conclusions concerning the differences between the 2 strains of mice across laboratories, remained in the heterogenised design. Heterogenised or homogenised designs were also similar in terms of consistency across laboratories, coverage probability, or proportion of accurate results. Systematic heterogenisation of experimenters failed to increase within-study variance above between-laboratory variation for behavioural or physiological outcomes [10].

Despite the large proportion of explained variation by “mice strain,” “laboratory,” or interaction between “strain and laboratory,” residual variance appeared to be a major source of variation in 6 out of 10 outcomes [10]. Most of the variance in the study came from unknown sources, leaving the causes of the variation open to conjecture and subsequent studies. The authors discussed approaches for future studies to identify known and unknown background factors that integrate uncontrolled variation in behavioural studies. In line with their suggestions, we envisioned some designs for studies pursuing to investigate systematic heterogenisation of animal studies in single- or multilaboratory settings (Fig 1). For example, in experiments performed uniquely on females like theirs [10], the estrous cycle could explain a meaningful portion of the unknown variance. Therefore, the estrous cycle, divided into 2 (luteal phase and follicular) or 4 phases (proestrous, estrous, metestrous, and diestrous), could be a heterogenisation factor in future investigations. In this hypothetical study, the outcomes of females synchronised in a particular estrous phase (homogeneous design) were compared to those of females at different estrous phases (heterogeneous design).

Considering behavioural outcomes specially, it would be interesting to know whether the sex of laboratory animals [2] or their behavioural strategies [4] could add a significant amount of variation to these studies. Female readouts in behavioural tests, mainly validated in male animals, may reflect a behavioural strategy to deal with stressors or novelty distinct from that of males [2]. Upcoming studies considering sex or behavioural strategy as a heterogenisation factor could estimate the consistency of outcomes within homogeneous and heterogeneous designs. Sex is often divided into 2 distinct categories (male and female). Behavioural strategies can be divided into artificial categories according to the theoretical background that researchers intend to investigate (e.g., active or passive behaviours). Experimental groups in the homogenised design would be homogeneous (e.g., single sex and single behavioural strategy), while in the heterogenised design, groups would be heterogeneous (e.g., mixture of sexes and mixture of behavioural strategies). Successful systematic heterogenisation would reduce between-study variation, favouring generalisation and keeping within-studies variability under acceptable levels.
